# Case report: Insulin desensitization as the only option for managing insulin allergy in a Sudanese patient

**DOI:** 10.3389/falgy.2023.1089966

**Published:** 2023-05-09

**Authors:** Rihan Said, Rayan Goda, Ihab B. Abdalrahman, Nahla H. H. Erwa

**Affiliations:** ^1^ Endocrinology Unit, Department of Medicine, Soba University Hospital, University of Khartoum, Khartoum, Sudan; ^2^ Clinical Immunology and Allergy Unit, Soba University Hospital, Khartoum, Sudan; ^3^ Department of Medicine, Soba University Hospital, University of Khartoum, Khartoum, Sudan; ^4^ Department of Microbiology, Faculty of Medicine, University of Khartoum, Khartoum, Sudan

**Keywords:** anaphylaxis, insulin allergy, desensitization, allergen immunotherapy, insulin desensitization

## Abstract

**Introduction:**

Allergic reactions to insulin have become very rare with the introduction of human insulin. Anaphylaxis is a life-threatening condition that results from immediate IgE-mediated hypersensitivity. Desensitization to human insulin was reported to control immediate hypersensitivity reactions to insulin. Here, we describe the history and challenges of managing our patient and the development of an insulin desensitization protocol in a resource-limited setup.

**Case Summary:**

A 42-year-old Sudanese woman with poorly controlled type 2 diabetes on maximum antidiabetic medications required insulin therapy to achieve reasonable glycemic control. She developed progressive and severe immediate hypersensitivity reactions to insulin, including anaphylaxis. Serum sample analysis demonstrated insulin-specific IgE antibodies. The patient's poor glycemic control and the need for breast surgery indicated insulin desensitization. A 4-day desensitization protocol was delivered in an ICU bed for close observation. Following successful desensitization and 24-h observation, our patient was discharged on pre-meal human insulin, which was tolerated well to the current date

**Conclusions:**

Although insulin allergy is rare, once encountered, it is very challenging in patients who have no other treatment options available. Different protocols for insulin desensitization are described in the literature; the agreed protocol was implemented successfully in our patient despite the limited resources.

## 1. Introduction

The introduction of recombinant human insulin has resulted in a marked reduction in allergic reactions to insulin (0.1%–3%) in comparison to those seen with the use of bovine and swine insulin in the past (10%–50%) (1–4). The more recent insulin analogs showed even fewer reactions (5). While impurities were the main cause of insulin-related allergies in the past, in the era of modern insulin analogs, allergic reactions can result from protamine sulfate, preservatives like cresol and phenol, or the insulin molecule itself (6, 7).

Type I (IgE-mediated), type III (Arthus-type), and type IV (T-cell-mediated) reactions are all implicated in insulin allergy; hence, the clinical presentations of such cases are variable (6). IgE-mediated reactions generally present with urticaria, angioedema, gastrointestinal symptoms, respiratory symptoms, and, less frequently, life-threatening anaphylaxis. These reactions are diagnosed based on a convincing history, with the symptoms occurring within 2 h of exposure to the depicted antigen. Confirmation of the diagnosis requires the detection of specific IgE antibodies against the specific allergen by skin prick tests, intradermal tests, or specific IgE detection in the serum or plasma of the patient. Treatment of IgE-mediated reactions involves using antihistamines and other medicines, such as leukotriene inhibitors and topical steroids. Systemic steroids have been used, but the evidence is slightly weak. Omalizumab is a monoclonal antibody that has been approved for use in some IgE-mediated conditions and may be used in difficult cases of insulin allergy (7).

## 2. Case report

**Figure 1:**
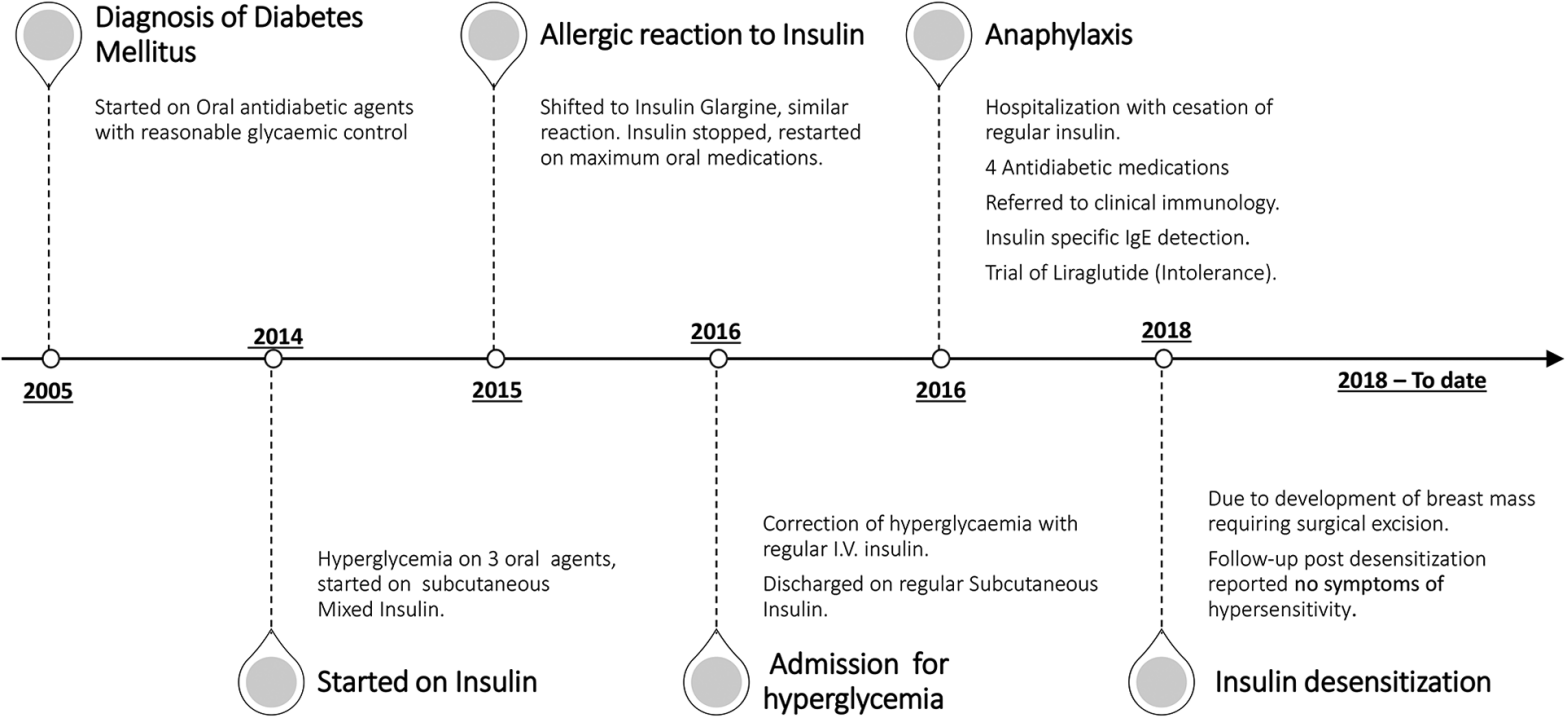
Timeline of the main events encountered during the patient's course of management and care.

A 42-year-old care worker who had had type 2 diabetes for 13 years was reviewed in the diabetes clinic for poor glycemic control. She was previously on oral antidiabetic medications with relatively reasonable control, but over the preceding year, her glycemic indices rose remarkably (HbA1c 11.5%) despite being on three antidiabetic agents (glimepiride, pioglitazone, and metformin). She was started on premixed insulin alongside metformin. During her follow-up 4 months later, she reported itching and a burning sensation starting 5 min following the insulin injection and resolving a few hours later. This had worsened gradually over the previous months. She was shifted to glargine alongside oral agents, but this also resulted in an allergic reaction: an induration that would last for a couple of days. Discontinuing her insulin soon resulted in symptomatic hyperglycemia, for which she was admitted and managed successfully in the emergency department with regular human insulin through direct intravenous access. She was discharged on regular insulin before meals and referred to the diabetes clinic. Unfortunately, within a few days, she developed anaphylaxis with laryngeal edema, for which she was resuscitated in the emergency department (Figure 1).

An urgent referral to clinical immunology was arranged, and a diagnosis of insulin allergy was made based on the mentioned history. The patient also reported urticarial reactions following exposure to certain local plants but has no allergy to latex or other medications. Her list of medications included atorvastatin, insulin, and oral antidiabetic medications. A trial of insulin Aspart with antihistamines (although tolerated well for a few weeks) also provoked a local allergic reaction. The diagnosis of insulin allergy, based on a history of type 1 hypersensitivity including anaphylaxis, was soon confirmed by detecting insulin-specific IgE in her serum (0.41 kU/L). With insulin being stopped, her antidiabetic agents were escalated to four medications by adding a dipeptidyl peptidase 4 inhibitor (DPP4i) (Figure 1). Her glycemic control continued to deteriorate, and on one occasion, she required intravenous insulin. This was done cautiously in the high dependency unit (HDU) while using antihistamines; no allergic reactions were observed during the 48-h stay.

Despite the unavailability of GLP-1 agonists locally, we managed to provide the patient with liraglutide as a last resort. Although this reflected positively on her subsequent blood glucose levels, she developed gastrointestinal side effects and was intolerant to the medication. After exhausting all the treatment options, diabetes and immunology teams decided to perform insulin desensitization as the next appropriate step, and a 4-day desensitization protocol was agreed on (Table 1). In the interim, our patient was seen in the surgical department for a breast lump, and surgical excision was advised once she achieved reasonable glycemic control.

**Table 1:** Insulin desensitization protocol.

Day	Insulin dose (IU)	Comments
1	0.00001 0.0001 0.001 0.01 0.1 0.5 1.0 2 4	Dose increments took place every 30 min Patient was closely monitored for late-phase reactions for 6 h following the last step of updosing
2	8 12	Doses were given 30 min apart The patient was monitored for hypoglycemia and late-phase reactions
3	16	Only one dose was given on day 3. Minor and brief induration was reported. Chlorpheniramine was given to avoid further progression of the reaction, and no further insulin doses were given
4	14 16	The patient agreed to continue with the desensitization after appropriate counseling and reassurance that she would be given 14 units before proceeding to the 16-unit step. Both doses were given before meals
5	14 14	Additional day to observe regular premeal insulin with antidiabetic No reactions were observed

An interdisciplinary meeting involving an endocrinologist, an immunologist, and an intensivist was held to explore the risks involved, agree on the desensitization protocol, allocate a spot in the intensive care unit (ICU), and provide in-service training on using the desensitization protocol. The protocol was based on the stepwise increase of insulin using subcutaneous injections of serial dilutions of *soluble insulin* to induce tolerance (Table 1). Loratadine 10 mg once daily was given as premedication 1 week before desensitization and continued throughout the process for 2 weeks. Montelukast 10 mg once daily was prescribed but was not tolerated by our patient.

On days 1 and 2, escalating insulin doses were given subcutaneously every 30 min until a total dose of 20 units was reached by the end of day 2 with no allergic reactions (Table 1). On days 3 and 4, premeal insulin was given; a minor induration was observed at the injection site when the dose was increased to 16 units on day 3. The patient also reported a mild itch in the upper chest, neck, and at the injection site. There was no visible skin rash. She was given intravenous antihistamine to avoid the progression of this reaction, and a decision was made to stop the desensitization at this stage. Further questioning revealed that the patient did not have a good night's sleep the previous night, which might have contributed to her symptoms. On day 4, the patient was well and willing to continue with the desensitization after appropriate counseling and reassurance. A modification of the protocol was made to restart on day 4 with a dose of 14 U before breakfast (09:30) and a prelunch insulin dose of 16 U (13:30). No further reactions were observed after both doses (Table 1). Following observation for 24 h on premeal insulin (on day 5), the patient was discharged on a total daily dose of 28 units (14 units before her two main meals), glimepiride 8 mg, vildagliptin 50 mg, and pioglitazone 30 mg (as her blood glucose remained high at 220–345 mg/dl). During subsequent follow-ups, the insulin dose was gradually increased, with a marked improvement in her glycemic control and without any allergic consequences; her other antidiabetic agents were discontinued. A few months later, mixed insulin (soluble/NPH) twice per day was introduced and tolerated well by the patient again. Four years following desensitization, our patient remains well, with reasonable glycemic control.

## 3. Discussion

The prevalence of people with diabetes who developed allergic reactions to human insulin has been reported to be less than 2% (8). Options for managing insulin allergy include stopping insulin, switching to a different type of insulin, and desensitization. Using insulin pumps (subcutaneous and intravenous) is reported in the literature to help insulin tolerance (9). While introducing newer antidiabetic agents, like GLP-1 agonists and SGLT2 inhibitors, in the management of patients with type 2 diabetes can help in achieving reasonable glycemic control, these may not be tolerated, accessible, or affordable for a sector of patients. Furthermore, insulin would be the only resort once glycemic control deteriorates despite maximum antidiabetic therapy. Introducing a GLP-1 agonist resulted in a marked improvement in glycemic control, but the drug was not tolerated by our patient. Switching to locally available insulin analogs was not successful in the presence of IgE antibodies against insulin, although this approach was successfully applied using glargine and lispro (10).

Our patient presented with anaphylaxis and urticarial reactions to subcutaneous insulin while tolerating it via the intravenous route. Reactions occurring through the subcutaneous route, as opposed to intravenously, might be attributable to the different ways in which insulin is presented to T cells via skin Langerhan's cells (11). Similarly, Asai et al. reported no symptoms following the intravenous administration of insulin (12). The same authors used an intravenous portable insulin pump to deliver insulin over a central line to induce immunologic tolerance in their patient, but a similar device was not available within our setting.

Omalizumab (an anti-IgE monoclonal antibody) has been used to induce tolerance to insulin in a number of cases (7). Pancreatic transplantation has been reported as a last resort. Both of these options were not feasible for our patient (13).

### 3.1. Desensitization

Allergen desensitization was introduced over 100 years ago by Noon, who succeeded in treating hay fever with the injection of grass extracts (14).

The standard method of desensitization involves the subcutaneous injection of increasing concentrations of the purified allergen extracts to induce tolerance to the injected allergen. Shorter protocols of desensitization (rush and ultrarush) are used in some cases for patients who are allergic to essential medicinal products (aspirin, antibiotics, insulin), where IgE hypersensitivity has been proven as a cause of patient reactions (15). Rapid drug desensitization was first successfully performed in the 1940s for penicillin allergy (16, 17).

With the encouraging reports of successful insulin desensitization using ultrarush protocols, this procedure was considered the best option for our patient (13, 18). The anaphylactic nature of her reactions to regular human insulin, the absence of adrenaline autoinjectors as rescue medication in Sudan, the patient's poor glycemic control, and the very limited resources made the decision to desensitize more pressing yet challenging. The MDT decision to desensitize the patient was made mainly on a clinical basis, with careful risk assessment and resource management. The specific IgE level was low according to the laboratory reference range and might have been indicative of mere exposure to insulin. Nevertheless, this result was interpreted in correlation with the patient's clinical presentation (19). The patient's insulin desensitization protocol was adopted from the literature and modified according to the available local facilities and the patient's condition. This protocol was tailored only toward soluble insulin, as it is essentially required during the management of diabetic and medical emergencies. Allergy skin tests were not performed due to insufficient equipment and technical preparation support. This limitation to the diagnosis was overcome through careful clinical assessment. The procedure was conducted in the intensive care unit for close monitoring over the admission course.

On reviewing the literature, some authors reported ineffective desensitization, while others reported short-term responses in some cases. However, our patient continued to tolerate insulin well 4 years after desensitization.

### 3.2. Challenges and lessons learned

Healthcare delivery in developing countries is affected by many constraints that manifest as a lack or limitation of services (20). Standard allergen desensitization setups are not readily available in Sudan, and there are no drug desensitization reports from the country. Indeed, this is the first report of insulin desensitization from Sudan. It saved the patient's life and the cost of treatment, had she traveled abroad.

Eminent surgery for her breast mass, poor glycemic index, and recurrent life-endangering reactions worked in favor of desensitization. The desensitization protocol was written by the immunologists based on the available literature and was reviewed by an experienced senior clinical immunologist from a different country, where allergen immunotherapy is routinely done.

One of the challenges was the readiness of the hospital to carry out this procedure. The enthusiasm of hospital management and staff helped overcome hurdles by opening an extra bed, which was staffed by locum ICU nurses, and arranging in-service training on the management of anaphylaxis, which facilitated the safe and smooth delivery of the procedure. Overall, insulin desensitization, in this case, was a very useful exercise that called for sensitizing our system to bridge existing gaps. It proved that the logistics are manageable and risk can be lowered by carefully planning and supervising the intervention. Our health delivery system must remain resilient to adapt to future challenges, and teamwork is key to success.

## 4. Conclusion

This case report describes the first insulin desensitization procedure carried out in Sudan within a limited-resource setting. Managing the patient was quite challenging due to her poor glycemic control, limited available alternatives, and the need for insulin to which she had multiple adverse reactions involving all available preparations. Implementing a care plan needed an interdisciplinary approach, through which successful insulin desensitization was achieved. This improved our patient's future prognosis despite limited resources and reflected positively on her outcome, with no adverse events to the current date.

## Data Availability

The original contributions presented in the study are included in the article; further inquiries can be directed to the corresponding author.
